# Prevalence and risk factors of hepatitis B virus infection in Zakho City, Kurdistan Region, Iraq; a population-based study

**DOI:** 10.1016/j.ijregi.2025.100703

**Published:** 2025-07-11

**Authors:** Nawfal R. Hussein, Ibrahim A. Naqid, Halder J. Abozait, Nashwan MR Ibrahim, Shakir A. Jamal, Brisik H. Rashad, Rijwan Azad Waisi, Dalia Ayhan Naji, Nadia Sulaiman Salih, Iman Salah Hassan, Parween Fadhil Ahmed, Nidar Loqman Khwasty, Marwa Faris Haji, Alina Abo Issa, Marwa Talib Abdulrazaq, Merdeen Muhammed Rasheed Morad, Zana Sidiq Mohammed Saleem, Dildar H. Musa

**Affiliations:** 1 Department of Biomedical Sciences, College of Medicine, University of Zakho, Zakho City, Iraq; 2 Department of Medicine, College of Medicine, University of Duhok, Duhok City, Iraq; 3 Department of Surgery, College of Medicine, University of Duhok, Duhok City, Iraq; 4 Department of Medical Education Development, College of Medicine, University of Zakho, Zakho City, Iraq

**Keywords:** Hepatitis B virus (HBV), Prevalence, Risk factors, Zakho City, Kurdistan Region, Iraq, Population-based study

## Abstract

**Background and objectives:**

Hepatitis B virus (HBV) infection remains a significant global health challenge, particularly in developing countries. Despite the availability of an effective vaccine, chronic HBV infection continues to cause substantial morbidity and mortality. This study aimed to determine the prevalence and associated risk factors of HBV infection in Zakho City, Kurdistan Region of Iraq.

**Methods:**

A population-based cross-sectional study was conducted on 26 February 2025 in Zakho City, Kurdistan Region, Iraq, using a multistage random sampling method. Data were collected from 386 participants through structured face-to-face interviews and serological testing for hepatitis B surface antigen (HBsAg) and anti-hepatitis B core immunoglobulin G antibody (HBcAb) using enzyme-linked immunosorbent assay.

**Results:**

The overall HBV positivity rate (HBcAb-positive) was 8%, with 7% indicating past infection (HBcAb-positive, HBsAg-negative) and 1% indicating chronic infection (HBcAb-positive and HBsAg-positive). In the multivariate analysis, HBV positivity was significantly associated with older age (*P* = 0.001) and male gender (*P* = 0.026). The highest prevalence (32.1%) was observed among participants aged ≥ 60 years.

**Conclusions:**

The findings demonstrate a moderate prevalence of past HBV infection and a relatively low rate of chronic carriers in Zakho city. Older age and male gender were significant predictors of HBV positivity. These results underscore the importance of continued public health efforts in HBV screening, early detection, and vaccination, particularly targeting older and at-risk populations.

## Highlights

• This study found an 8% hepatitis B virus (HBV) (hepatitis B core antibody-positive) rate and 1% chronic infection.
• HBV positivity was significantly associated with older age and male gender.
• Prevalence peaked at 32.1% in those aged ≥60.
• Highlight need for targeted HBV screening and vaccination efforts.

## Introduction

Infection with the hepatitis B virus (HBV) is a major global health issue. Around 296 million people were chronically infected with the virus in 2023 [1]. Chronic hepatitis B can lead to serious complications such as cirrhosis, liver failure, and hepatocellular carcinoma, and mortality from hepatitis B is rising, with roughly 1.1 million deaths attributed to the disease in 2022 [2]. HBV is a blood-borne virus transmitted through exposure to body fluids during sexual intercourse, intravenous drug use, dialysis, tattooing, needle-stick injuries, and perinatal vertical transmission during delivery [3,4]. The hepatitis B vaccine is more than 95% effective in preventing infection and is usually delivered in three doses after birth [5]. The prevalence of hepatitis B varies significantly between countries and within regions of each country, ranging from 5.8% in Africa to 0.5% in the region of the Americas, with the vast majority of cases residing in developing countries [2]. Following the ambitious plan of the World Health Organization (WHO) to eliminate viral hepatitis as a public health problem by 2030 [2], updated data regarding the burden and risk factors of hepatitis B holds significant value for planning and delivering targeted interventions to each region and helps redirect the resources to areas in need. Screening at-risk individuals is necessary to prevent the spread of infection because only 13% of people living with chronic infection had been diagnosed by 2022 [2]. While previous studies in Iraq have reported prevalence rates ranging from approximately 1% in the northern regions to 3.5% in the southern regions [[6], [7], [8], [9]]. However, these studies are outdated and primarily based on selected populations, such as blood donors or patients, which limits their generalizability to the broader community. No population-based study has yet been conducted in Zakho City. This lack of recent, representative data highlights the need for updated research to better understand the true burden and risk factors of hepatitis B in the general population. This study aimed to determine the prevalence and risk factors of hepatitis B infection in Zakho City, Kurdistan Region of Iraq.

## Materials and methods

A population-based study was conducted on February 26, 2025, in Zakho City, Kurdistan Region of Iraq. A multistage sampling method was used to select the participants. Initially, we used artificial intelligence to randomly select six districts out of those in Zakho City. Then, a random number generator was used to randomly select households. To ensure that the sample size was maximized, a population proportion of *P* = 0.5 was assumed because the true proportion was unknown. The initial sample size was determined to be 384 participants by applying the formula for an infinite population.

### Data collection and blood samples

A structured questionnaire was used to collect data through face-to-face interviews, including demographic and clinical data, and 386 participants were included in the study. A 5-cc syringe and needle were used to collect 5 mL of blood from each participant. These samples were centrifuged at 1500 rpm for 3 minutes to separate sera from blood and were then immediately tested for hepatitis B surface antigen (HBsAg) and anti-hepatitis B core antibody (HBcAb) or kept frozen at 20°C until the tests were performed later.

### Enzyme-linked immunosorbent assay

To detect HBsAg and HBcAb immunoglobulin G, an enzyme-linked immunosorbent assay test, specifically a commercial LIAISON XL diagnostic system (USA), was used following the manufacturer’s instructions. First, specific monoclonal antibodies (anti-HBsAg and anti-HBcAb) were fixed to the surface of the microwells. Sera from the study participants were added to the microwell, and secondary monoclonal antibodies conjugated with horseradish peroxidase were added. Unbound serum proteins and the horseradish peroxidase conjugate were then washed off. The substrate was added after blocking the enzymatic reaction, and the optical density was measured by an enzyme-linked immunosorbent assay reader.

### Statistics

Data were organized, encoded, and cleared using Microsoft Excel Professional Plus 2024 and then exported to IBM SPSS Statistics Version 25 software for all computational analysis. Descriptive statistics were performed to calculate numbers, percentages, and means ± standard deviation. The independent sample t-test was used to test for association with age. Univariate and multivariate analysis was performed using binary logistic regression to calculate the crude and adjusted odds ratios, *P*-values, and 95% confidence intervals. Statistical significance was set at *P*-value <0.05.

## Results

### Demographic data

Of the 386 participants included in this study, 64.5% were men and 35.5% were women. The mean age of the study participants was 33.6 ± 13.4 years, with nearly half (46.6%) under the age of 30 years. The majority of participants (93.8%) were from urban areas, and 50.1% were married. The demographic and clinical data are summarized in Table 1.

**Table 1:** Characteristics for study participants.

Characteristics	Number	Percentage
Age (years)		
≤29	180	46.6%
30-44	132	34.2%
45-59	46	11.9%
≥60	28	7.3%
Gender		
Female	137	35.5%
Male	249	64.5%
Residency		
Rural	24	6.2%
Urban	362	93.8%
Married		
No	158	40.9%
Yes	228	59.1%
History of jaundice		
No	332	86%
Yes	54	14%
History of hepatitis B		
No	375	97.2%
Yes	11	2.8%
Family history of hepatitis B		
No	366	94.8%
Yes	20	5.2%
Hepatitis B vaccination		
No	342	88.6%
Yes	44	11.4%
History of the surgical operation		
No	217	56.2%
Yes	169	43.8%
History of transfusion		
No	315	81.6%
Yes	71	18.4%
History of the dental procedure		
No	111	28.8%
Yes	275	71.2%
History of over-the-counter injections		
No	181	46.9%
Yes	205	53.1%
History of tattooing		
No	347	89.9%
Yes	39	10.1%

### Hepatitis B positivity

The prevalence of HBV positivity (positive HBcAb) in this study was 8% (31/386). The prevalence of prior infection with HBV (positive HBcAb with negative HBsAg) was 7% (27/386), while the prevalence of chronic HBV carriers (positive HBcAb and HBsAg) was 1% (4/386). All calculations for risk factors were based on HBV positivity.

### Risk factors for hepatitis B virus positivity

The mean age of HBV-positive participants was 12.8 years higher than that of HBV-negative participants, according to the *t*-test (95% CI: 6.77–18.82; *P* = 0.001). Univariate analysis showed that older age and being married were significant predictors of HBV positivity (*P* = 0.045 for ages 45–59 years, *P* = 0.001 for 60 years or more, and *P* = 0.035 for being married). Multivariate analysis showed that older age and male gender were significant predictors of HBV positivity (*P* = 0.023 for ages 30–45 years, *P* = 0.008 for 45–59 years, *P* = 0.001 for 60 years or more, and *P* = 0.026 for gender). Table 2 shows the statistical analysis.

**Table 2:** Risk factors for HBV infection.

Factors	HBV negative	HBV positive	Crude OR (95% CI)	*P*-value	Adjusted OR (95% CI)	*P*-value
**Age (years)**						
≤29	174 (96.7%)	6 (3.3%)	1		1	
30-44	121 (9.7%)	11 (8.3%)	2.64 (0.95-7.32)	0.063	4.74 (1.24-18.13)	0.023
45-59	41 (89.1%)	5 (10.9%)	3.54 (1.03-12.16)	0.045	10.56 (1.87-59.64)	0.008
≥60	19 (67.9%)	9 (32.1%)	13.74 (4.41-42.8)	0.001	45.43 (7.98-258.65)	0.001
**Gender**						
Female	131 (95.6%)	6 (4.4%)	2.44 (0.97-6.1)	0.057	3.209 (1.15-8.97)	0.026
Male	224 (90%)	25 (10%)				
**Residency**						
Urban	334 (92.3%)	28 (7.7%)	1.704 (0.48-6.07)	0.411	3.277 (0.76-14.14)	0.112
Rural	21 (87.5%)	3 (12.5%)				
**Married**						
No	151 (95.6%)	7 (4.4%)	2.54 (1.07-6.04)	0.035	0.584 (0.17-2.05)	0.402
Yes	204 (89.5%)	24 (10.5%)				
**History of jaundice**						
No	309 (93.1%)	23 (6.9%)	2.336 (0.99-5.53)	0.054	1.917 (0.63-5.82)	0.251
Yes	46 (85.2%)	8 (14.8%)				
**History of hepatitis B**						
No	346 (92.3%)	29 (7.7%)	2.65 (0.55-12.85)	0.226	3.904 (0.55-27.89)	0.175
Yes	9 (81.8%)	2 (18.2%)				
**Family history of B hepatitis**						
No	337 (92.1%)	29 (7.9%)	1.29 (0.285-5.84)	0.74	1.426 (0.247-8.24)	0.692
Yes	18 (90%)	2 (10%)				
**Hepatitis B vaccination**						
No	317 (92.7%)	25 (7.3%)	2.002 (0.77-5.19)	0.153	1.215 (0.36-4.15)	0.755
Yes	38 (86.4%)	6 (13.6%)				
**History of the surgical operation**						
Yes	157 (92.9%)	12 (7.1%)	1.255 (0.59-2.66)	0.553	1.906 (0.75-4.84)	0.174
No	198 (91.2%)	19 (8.8%)				
**History of transfusion**						
Yes	67 (94.4%)	4 (5.6%)	1.57 (0.53-4.64)	0.414	2.22 (0.68-7.23)	0.186
No	288 (91.4%)	27 (8.6%)				
**History of the dental procedure**						
No	103 (92.8%)	8 (7.2%)	1.175 (0.51-2.71)	0.705	0.996 (0.38-2.62)	0.993
Yes	252 (91.6%)	23 (8.4%)				
**History of over-the-counter injections**						
Yes	189 (92.2%)	16 (7.8%)	1.067 (0.51-2.23)	0.862	0.761 (0.323-1.79)	0.532
No	166 (91.7%)	15 (8.3%)				
**History of tattooing**						
No	322 (92.8%)	25 (7.2%)	2.342 (0.9-6.12)	0.082	1.773 (0.53-5.93)	0.352
Yes	33 (84.6%)	6 (15.4%)				

CI, confidence interval; HBV, hepatitis B virus; OR, odds ratio.

A *P*-value <0.05 is considered statistically significant.

## Discussion

Hepatitis B remains a major public health threat, particularly in developing countries; however, its prevalence varies geographically [2,10]. In the present study, the overall prevalence of HBV positivity was 8%. Among these, 7% had evidence of past infection (HBcAb-positive and HBsAg-negative), while only 1% were chronically infected (both HBcAb- and HBsAg-positive). The prevalence of chronic hepatitis B infection in this study (1%) is lower than the global prevalence reported in 2022 (3.2%) [2]. Other studies conducted in the region have reported similar findings, with hepatitis B prevalence rates ranging from 0.54% to 1.37% [[10], [11], [12], [13]]. In neighboring countries, prevalence rates vary from 1.7% in Saudi Arabia and 3% in Turkey to 3.4% in Iran [[14], [15], [16]]. Vaccination coverage has increased significantly since 2000, reaching 90% in Iraq and exceeding 97% in Saudi Arabia, Turkey, and Iran [17].

This observed decline in prevalence may also be attributed to the implementation of updated infection control protocols and comprehensive screening programs. These efforts have likely contributed to reducing transmission by facilitating early detection and minimizing exposure in both healthcare and community settings.

In this study, the mean age of HBV-positive participants was 45.3 years, which was 12.8 years higher than that of HBV-negative participants, as determined by the t-test (95% CI: 6.77-18.82; *P* = 0.001). Both univariate and multivariate logistic regression analyses demonstrated a significant association between age and HBV positivity, with the highest prevalence (32.1%) observed among participants aged 60 years or older. Our findings agree with the results from regional and Iranian studies [10,11,18]. The increased prevalence in older individuals may reflect a cohort effect, where exposure to HBV occurred more frequently in earlier decades before the widespread adoption of vaccination and infection control measures. Men were more likely to have HBV infection than women (10% compared to 4.4%). This association was significant once the confounding factors were controlled in the multivariate analysis. Our results are in agreement with a study from Turkey [19] but contradict a regional paper in which women were more likely to have hepatitis B [20]. A regional study and an Iranian study showed no association between gender and hepatitis B [10,18]. High-risk behaviors such as intravenous drug use or illegitimate sex may have contributed to the higher prevalence of HBV among men in this study. There was no statistically significant association between rural residency and HBV infection in this study. These results are similar to those of some studies [13,18], but other studies have found such an association [11,20]. Married participants were more likely to have HBV infection than unmarried individuals (10.5% vs 4.4%), a pattern consistent with findings from several studies [10,18,20]. This association may be explained by the sexual transmission of the virus, which is more likely within marital relationships. Additionally, marriage is often associated with older age, which may confound the relationship. Notably, the association lost significance in the multivariate analysis, suggesting that age or other factors may be the true underlying contributors. No prior history of jaundice or hepatitis B was associated with HBV positivity in this study, possibly due to recall bias and the small sample size of this study. Infection with hepatitis B in adults is cleared in more than 95% of cases and has vague symptoms that might be overlooked by the general population [1]. A study from Turkey found a significant association between HBV infection and having a family member with hepatitis B, such as a spouse or parents [19]; in contrast, we have not found such an association. Similarly, although vaccination has established effectiveness and is widely incorporated into the Expanded Program on Immunization [5], we could not detect an association between HBV positivity and history of vaccination. This may be due to recall bias, especially among older participants who may not accurately remember their vaccination status. Additionally, incomplete vaccination, delays in receiving doses, or lack of documentation could contribute to the observed lack of association. In this study, there was no significant association between HBV positivity and a personal history of surgery, dental procedure, and over-the-counter injections, transfusion with blood or blood products, or history of tattooing. These results are similar to those of other studies conducted regionally and in neighboring countries [[10], [11], [12], [13],^18^,^20^]. The only exceptions were a significant correlation between HBV positivity with a history of tattooing in two studies [10,11]; and dental procedure in one study [12]. The change in the distribution of risk factors and the lack of significant association are likely due to multiple factors such as widespread vaccination, strict preventive measures taken prior to and during surgical operations and dental procedures, screening of blood used for transfusion, and the use of sterile equipment for tattooing and injections.

## Conclusion

This study provides important insights into the epidemiology and risk factors of HBV infection in Zakho City, located in the Kurdistan Region of Iraq. The overall HBV prevalence was 8%, with chronic infections accounting for only 1%, a figure lower than both global and regional averages. This relatively low rate may reflect the success of the universal vaccination program and improvements in infection control strategies.

The study highlights a higher prevalence of HBV among older individuals, particularly those aged 60 years and above, and among males, possibly due to increased exposure to high-risk behaviors. A significant association was also observed between marital status and HBV positivity; however, this relationship appeared to be influenced by age. Although no significant association was found with self-reported vaccination history, the overall decline in HBV transmission supports the effectiveness of vaccination programs and infection control efforts.

Based on these findings, it is recommended to strengthen public health campaigns to raise awareness, enhance the accuracy of vaccination records, and continue robust infection control measures to sustain the reduction in HBV prevalence.

## Declarations of competing interest

The authors have no competing interests to declare.

## References

[1] Hsu Y.C., Huang D.Q., Nguyen MH. (2023). Global burden of hepatitis B virus: current status, missed opportunities and a call for action. Nat Rev Gastroenterol Hepatol.

[2] Songtanin B., Chaisrimaneepan N., Mendóza R., Nugent K. (2024). Burden, outcome, and comorbidities of extrahepatic manifestations in hepatitis B virus infections. Viruses.

[3] World Health Organization (2024).

[4] Li C., Wei C., Yang X. (2024). Hepatitis B virus: modes of transmission, immune pathogenesis, and research progress on therapeutic vaccines. Explor Dig Dis.

[5] di Filippo, Villa D., Navas MC. (2023). Vertical transmission of hepatitis B virus-an update. Microorganisms.

[6] Pattyn J., Hendrickx G., Vorsters A., Van Damme P. (2021). Hepatitis B vaccines. J Infect Dis.

[7] S Khalil A., Abdulkareem W.L., Hussein NR. (2018). The effectiveness of post-exposure prophylaxis in infants born to hepatitis B virus positive mothers in the Kurdistan Region. Iraq. Int J Infect.

[8] Hussein N.R., Saleem Z.S., Ibrahim N.M., Saleh Assafi M., Daniel S. (2019). The prevalence of HBV infection in renal transplant recipients and the impact of infection on graft survival. Acta Med Iran.

[9] Al-Nafakh R.T., Al-Nafakh R.T., Al-Sherees H.A.A., Al-Charrakh A.H. (2019). Seroprevalence of HBV, HCV, and HIV among blood donors in main blood bank in Najaf Province. Iraq. Indian J Public Health Res Dev.

[10] Al-Rubaye A., Tariq Z., Alrubaiy L. (2016). Prevalence of hepatitis B seromarkers and hepatitis C antibodies in blood donors in Basra, Iraq. BMJ Open Gastroenterol.

[11] Al Juboury A., Salih H., Al-Assadi M., Ali A.M. (2010). Seroprevalence of hepatitis B and C among blood donors in Babylon Governorate-Iraq. Med J Babylon.

[12] European Centre for Disease Prevention and Control (2024).

[13] Hussein N.R., Musa D.H., Hawezy D.J., Ahmed F.M.R., Khalid F.K., Naqid I.A., Assafi MS. (2021). A study on the prevalence and the risk factors of hepatitis B virus infection in Kurdistan Region, Iraq: a multicenter study. J Contemp Med Sci.

[14] Khalid F.K., Rasheed N.A., Hussein N.R., Naqid IA. (2022). A study of HBV infection and its risk factors in pregnant women in Zakho city. Iraq. PLoS One.

[15] R Hussein NR. (2018). Risk factors of hepatitis B virus infection among blood donors in Duhok City, Kurdistan Region, Iraq. Caspian J Intern Med.

[16] Rasheed Hussein N., Mohamad Haj S., Amin Almizori L., Ahmed Taha A. (2016). The prevalence of hepatitis B and C viruses among blood donors attending blood bank in Duhok, Kurdistan Region, Iraq. Int J Infect.

[17] Abaalkhail F.A., Al-Hamoudi W.K., Khathlan A., Alghamdi S., Alghamdi M., Alqahtani S.A. (2021). SASLT practice guidelines for the management of hepatitis B virus – an update. Saudi J Gastroenterol.

[18] Yekenkurul D., Gürbüz A.R., Ince N., Çalışkan E. (2024). Prevalence of HBV, HCV, HIV and effect on clinical course in COVID-19 patients. J Health Sci.

[19] Mokhayeri H., Hasanvand B., Birjandi M., Mirzaei H., Sasaei P., Zamani S. (2024). Prevalence of HIV, HBV, and HCV infections and high-risk behaviors among women referred to drop-in centers in Lorestan Province, Western Iran. Arch Razi Inst.

[20] Hussein N.R., Abozait H.J., Naqid I.A., Ibrahim N.M., Khalid F.K., Musa D.H. (2025). Risk factors of hepatitis B virus infection in the Kurdistan Region of Iraq: a cross-sectional study. Mediterr J Hematol Infect Dis.

